# Proteomic Changes in Human Sperm During Sequential *in vitro* Capacitation and Acrosome Reaction

**DOI:** 10.3389/fcell.2019.00295

**Published:** 2019-11-20

**Authors:** Judit Castillo, Orleigh Adeleccia Bogle, Meritxell Jodar, Forough Torabi, David Delgado-Dueñas, Josep Maria Estanyol, Josep Lluís Ballescà, David Miller, Rafael Oliva

**Affiliations:** ^1^Molecular Biology of Reproduction and Development Research Group, Institut d’Investigacions Biomèdiques August Pi i Sunyer (IDIBAPS), Fundació Clínic per a la Recerca Biomèdica, Department of Biomedical Sciences, Faculty of Medicine and Health Sciences, Universitat de Barcelona, Barcelona, Spain; ^2^LIGHT Laboratories, Leeds Institute of Cardiovascular and Metabolic Medicine, University of Leeds, Leeds, United Kingdom; ^3^Proteomics Unit, Scientific and Technical Services, Universitat de Barcelona, Barcelona, Spain; ^4^Clinic Institute of Gynaecology, Obstetrics and Neonatology, Hospital Clínic, Barcelona, Spain; ^5^Biochemistry and Molecular Genetics Service, Hospital Clínic, Barcelona, Spain

**Keywords:** sperm, spermatozoa, capacitation, acrosome reaction, proteomics, mass spectrometry, TMT labeling

## Abstract

The male gamete is not completely mature after ejaculation and requires further events in the female genital tract to acquire fertilizing ability, including the processes of capacitation and acrosome reaction. In order to shed light on protein changes experienced by the sperm cell in preparation for fertilization, a comprehensive quantitative proteomic profiling based on isotopic peptide labeling and liquid chromatography followed by tandem mass spectrometry was performed on spermatozoa from three donors of proven fertility under three sequential conditions: purification with density gradient centrifugation, incubation with capacitation medium, and induction of acrosome reaction by exposure to the calcium ionophore A23187. After applying strict selection criteria for peptide quantification and for statistical analyses, 36 proteins with significant changes in their relative abundance within sperm protein extracts were detected. Moreover, the presence of peptide residues potentially harboring sites for post-translational modification was revealed, suggesting that protein modification may be an important mechanism in sperm maturation. In this regard, increased levels of proteins mainly involved in motility and signaling, both regulated by protein modifiers, were detected in sperm lysates following incubation with capacitation medium. In contrast, less abundant proteins in acrosome-reacted cell lysates did not contain potentially modifiable residues, suggesting the possibility that all those proteins might be relocated or released during the process. Protein-protein interaction analysis revealed a subset of proteins potentially involved in sperm maturation, including the proteins Erlin-2 (ERLIN2), Gamma-glutamyl hydrolase (GGH) and Transmembrane emp24 domain-containing protein 10 (TMED10). These results contribute to the current knowledge of the molecular basis of human fertilization. It should now be possible to further validate the potential role of the detected altered proteins as modulators of male infertility.

## Introduction

Mammalian fertilization relies on the ability of spermatozoa to penetrate through the zona pellucida (ZP) and fuse with the oolemma. Testicular spermatozoa, however, although morphologically mature, lack this essential capacity and additional post-gonadal stages of maturation are required in both the male and the female reproductive tracts. After being released into the lumen of the seminiferous tubules, testicular spermatozoa are sequentially modified throughout their transit in the epididymis, acquiring forward progressive motility (Dacheux and Dacheux, 2014). However, maturation is not completed until spermatozoa undergo capacitation during their journey through the female genital tract (Visconti et al., 1998; Stival et al., 2016; Puga Molina et al., 2018a). Sperm are only capable of successfully fertilizing the oocyte after completing this essential post-ejaculatory step, which is a prerequisite to the acrosome reaction (Barros, 1967; Gaddum and Blandau, 1970; Visconti and Kopf, 1998; Visconti et al., 1998, 2011; De Jonge, 2005; Liu et al., 2007).

Sperm capacitation is a recognized phenomenon associated with acquisition of hyperactive motility comprising a complex set of highly regulated molecular and physiological events. As mature spermatozoa are no longer engaged in nuclear gene expression (Oliva, 2006; Jodar et al., 2013), understanding the role of protein modifiers, cell-cell communication and signaling pathways is essential (Baldi et al., 1996; Halet et al., 2003; De Jonge, 2005; Visconti et al., 2011; Buffone et al., 2012; Signorelli et al., 2012; Cuasnicú et al., 2016; Barrachina et al., 2018; Torabi et al., 2017b). For instance, many studies have correlated capacitation with an increase in cholesterol efflux from the sperm membrane (Visconti et al., 2002; Gadella et al., 2008) that modulates intracellular levels of calcium and bicarbonate ions (Visconti et al., 2011; Battistone et al., 2013; Li et al., 2014; Puga Molina et al., 2018a). As a consequence, other changes during capacitation include the hyperpolarization of the sperm plasma membrane (Puga Molina et al., 2017, 2018b), and the enhanced activity of protein kinases, phosphatases and acetylases (Ficarro et al., 2003; Ickowicz et al., 2012; Battistone et al., 2014; Sati et al., 2014; Wang J. et al., 2015; Puga Molina et al., 2017; Ritagliati et al., 2018). In addition, protein regulation through redox signaling has been proposed as one of the critical properties of sperm capacitation (O’Flaherty, 2015). All these changes occur prior to the acrosome reaction, a calcium-dependent process leading to a sequential release of the acrosomal contents (Buffone et al., 2008), which includes hydrolytic enzymes, such as acrosin and hyaluronidase, that allow sperm penetration through the ZP and subsequent fusion with the oocyte membrane (Brucker and Lipford, 1995; Patrat et al., 2000; Ickowicz et al., 2012; De Jonge and Barratt, 2013; Petit et al., 2013; Sánchez-Cárdenas et al., 2014; Singh and Rajender, 2015; Ito and Toshimori, 2016; Torabi et al., 2017a). The role of kinases and tyrosine phosphorylation has been also established as essential for sperm to correctly undergo acrosome reaction (Ickowicz et al., 2012; Breitbart and Finkelstein, 2015).

The analysis of protein profiles of lysates obtained from human sperm cell has revealed a complex set of proteins involved in processes culminating in oocyte fertilization, including capacitation, the acrosome reaction, oocyte penetration and sperm-oocyte fusion (Castillo et al., 2018). Indeed, capacitation is the event most studied by the application of quantitative proteomic strategies in human (Ficarro et al., 2003; Secciani et al., 2009; Wang J. et al., 2015; Yu et al., 2015; Hernández-Silva et al., 2019) as well as in model species, such as mice (Baker et al., 2010), boars (Bailey et al., 2005; Kwon et al., 2014), bulls (Park et al., 2012) and buffalo (Jagan Mohanarao and Atreja, 2011; Hou et al., 2019). Recently, Hernández-Silva et al. (2019) approached the proteomic study of human sperm capacitation from the alternative perspective of the seminal fluid components bathing sperm during and following ejaculation. Interestingly, they found a group of seminal fluid derived proteins attached to the sperm surface which inhibits the progress of capacitation by negatively affecting sperm hyperactivation and protein tyrosine phosphorylation (Hernández-Silva et al., 2019).

The study of the sperm protein composition is contributing not only to increase the knowledge of the male gamete structure and cargo, but also of past and future events related to sperm development and oocyte fertilization, respectively (Amaral et al., 2014a; Codina et al., 2015; Castillo et al., 2018). Mass spectrometry (MS) strategies are rapidly evolving, and novel approaches are currently available that could provide further insights into the maturation of the ejaculated sperm (Codina et al., 2015). Also, identifying protein changes in abundance and post-translational modifications during the different steps of sperm maturation is a good strategy to find candidates that could act as male infertility biomarkers with predictive, diagnostic and prognostic potentials (Rahman et al., 2013; Amaral et al., 2014a; Jodar et al., 2017). However, while the involvement of sperm proteins in ZP binding has been explored by proteomics (Redgrove et al., 2011; Petit et al., 2013), the sperm protein profile after the acrosomal exocytosis has not yet been addressed by MS. In the current study we employed a quantitative proteomics strategy based on isotopic peptide labeling using tandem mass tags (TMT), protein identification by liquid chromatography followed by tandem MS (LC-MS/MS), and strict and robust quantification and statistical analysis to evaluate changes in ejaculated spermatozoa after incubation in capacitation medium and calcium ionofore-induced acrosome reaction, aiming to improve the knowledge of the molecular mechanisms leading to post-ejaculation sperm maturation from a proteomic perspective.

## Materials and Methods

### Biological Material and Sample Collection

Human semen samples were obtained from 3 donors of proven fertility attending the Assisted Reproduction Unit (FIVclinic) at the Clinic Institute of Gynaecology, Obstetrics and Neonatology, from the Hospital Clínic, Barcelona, Spain. The sample size was calculated by considering a balance between (1) a sufficient number of samples to statistically evaluate the significance of the resulting data, and (2) the maximum amount of analytical samples that can be analyzed by quantitative proteomics using a unique set of Tandem Mass Tag^TM^ 6-plex (TMTsixplex^TM^) Reagents. The ejaculates were collected by masturbation into sterile containers following a minimum of 3 days abstinence. Semen was allowed to liquefy at room temperature and an initial evaluation of the seminal parameters was taken for concentration and motility assessment using the automatic semen analysis system CASA (Computer Assisted Semen Analysis; Proiser, Paterna, Spain). Additional parameters such as sperm variability and morphology were also assessed, using 0.5% (w/v) Eosyn Y and Diff-quick^TM^ staining, respectively. All three ejaculates showed normal parameters according to the limits established by the World of Health Organization (WHO) (World Health Organization, 2010) and were classified as normozoospermic semen samples (Supplementary Table S1).

All samples were used in accordance with the appropriate ethical guidelines and Internal Review Board, and the biological material storing and processing was approved by the Clinical Research Ethics Committee of the Hospital Clínic of Barcelona. Written informed consents were obtained from all donors in accordance with the Declaration of Helsinki.

### Sperm Preparation

**FIGURE 1:**
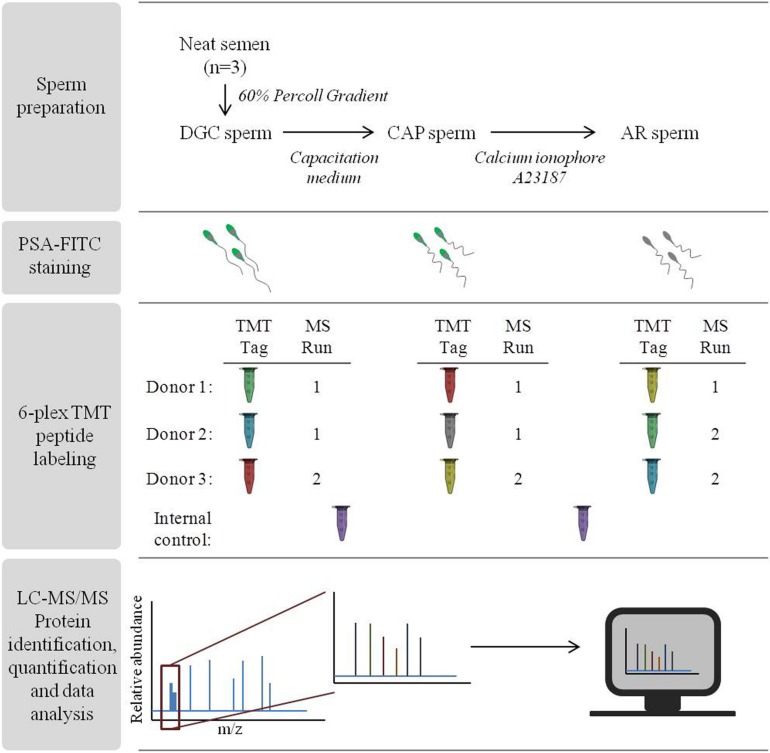
Overall workflow of the experimental procedures. Steps followed for the identification of sperm protein alterations with TMT-LC-MS/MS after consecutive *in vitro* incubation with capacitation medium followed by induction of the acrosome reaction.

Each ejaculate was processed under three sequential conditions (Figure 1): density-gradient centrifugation (DGC sperm), incubation in capacitation medium (CAP sperm) and incubation with the calcium ionophore A23187 to *in vitro* induce the acrosome reaction (AR sperm), following well-established protocols described elsewhere (World Health Organization, 2010; De Jonge and Barratt, 2013) with some minor modifications.

#### Density-Gradient Centrifugation

Spermatozoa were purified using a 60% Percoll^®^ (Sigma-Aldrich, St Louis, MO, United States) gradient equilibrated with Hepes-buffered Ham’s F10 medium (Gibco, Life Technologies, United Kingdom) supplemented with sodium bicarbonate (0.2% NaHCO3; w/v; Merck, Darmstadt, Germany), sodium pyruvate (0.003% C_3_H_3_NaO_3_; w/v; Sigma-Aldrich) and sodium DL lactate solution (0.36% v/v, Sigma-Aldrich). In order to ensure the complete elimination of the gradient material, the recovered sperm cells were washed twice with supplemented Hepes medium and centrifuged at 400 *g* for 10 min. This procedure allowed the purification of the samples from cells other than sperm while maintaining proportions of sperm subpopulations similar to the native semen sample (Mengual et al., 2003; Zhao et al., 2007; Wang G. et al., 2013). The sperm purification efficiency was checked using phase-contrast microscopy. All purified sperm samples contained <1% of potential contaminating cells. For each sample, an aliquot of 20 million of DGC sperm was taken for subsequent procedures of protein solubilization, while the remaining material was subjected to incubation with capacitation medium.

#### Sperm Incubation With Capacitation Medium

Capacitation medium consisted of Hepes-buffered Ham’s F10 medium supplemented with 3.5% of bovine serum albumin (BSA; Sigma-Aldrich). DGC sperm were incubated in 1 ml of capacitation medium for each 10 million of sperm, for 3 h at 37°C with constant rotation. Aliquots of 20 million of CAP sperm from each biological replicates were taken for protein solubilization and the remaining material were used for the incubation with the calcium ionophore A23187.

#### Sperm Incubation With the Calcium Ionophore A23187 to Induce the Acrosome Reaction

To induce the acrosome reaction, 10 μL of the calcium ionophore A23187 (Sigma-Aldrich, stock solution 1.0 mM in DMSO) was added to approximately 10 million CAP sperm to get a final concentration of 10 μM, following WHO recommendations (World Health Organization, 2010). Spermatozoa were then incubated for 15 min at 37°C. Negative control aliquots were incubated with DMSO (vehicle control) for an equal amount of time (De Jonge and Barratt, 2013). Subsequently, 70% ethanol was added to stop the reaction and the spermatozoa were recovered by centrifugation at 400 *g* for 20 min (World Health Organization, 2010). The viability of the cells was monitored by eosin staining (World Health Organization, 2010). For each replicate, an aliquot of 20 million of AR sperm was used for protein solubilization.

### *Pisum sativum* Agglutinin – Fluorescein Isothiocyanate (PSA-FITC) Labeling

The acrosome reaction was monitored by assessing the integrity of the sperm acrosome vesicle by the PSA-FITC labeling method (World Health Organization, 2010) (Figure 1). This fluorescent probe binds to the alpha-methyl mannose and labels the acrosomal content of sperm (Benoff et al., 1993). Briefly, 10 μl of each DGC, CAP, AR sperm and DMSO negative control (3 donors) were smeared on microscope slides in duplicates and were allowed to air dry. The slides were then fixed in 95% (v/v) ethanol for 30 min, and subsequently immersed in 25 μg/ml PSA-FITC staining solution, for 2 h at 4°C. The slides were washed in distilled water, air-dried and covered with mounting medium containing DAPI (Vectashield^®^, Vector Laboratories, Burlingame, CA, United States). The slides were visualized using a BX50 microscope (Olympus, Hamburg, Germany) equipped with a triple band pass filter, and images were acquired with an Olympus DP71 camera. The sperm acrosome status was categorized as: (1) intact acrosome (more than half of the sperm head with bright and uniform fluorescence); (2) reacted acrosome (a band of fluorescence localized to the equatorial segment or no fluorescing stain at all in the acrosome region); and (3) partially intact acrosome (all other sperm cells) (World Health Organization, 2010; De Jonge and Barratt, 2013). A minimum of 200 sperm were counted per slide and the mean of the two slides per sample represented the final value. Differences between the sperm treatments were evaluated by repeated measures ANOVA with Holm–Sidak correction. *P*-values < 0.05 were considered significant. Determination of the efficiency of the induction of acrosome reaction was also used as an indirect measure of efficiency on sperm capacitation (Torabi et al., 2017a).

### Protein Solubilization and Quantification

From each sperm preparation (DGC, CAP and AR sperm; 3 donors), proteins were solubilized by incubating 20 million spermatozoa with 50 μL of lysis buffer containing 2% SDS and 1 mM Phenylmethylsulfonyl fluoride (PMSF), for 30 min at room temperature with constant gentle shaking. Lysates were centrifuged at 16,000 *g* for 10 min at 4°C. Proteins in the soluble fraction were quantified using the BCA method (Thermo Fisher Scientific, Rockford, IL, United States), following manufacturer’s recommendations.

### Sperm Peptide Isotopic Labeling (TMT 6-Plex)

A total of 9 protein extracts were used for the proteomic study, corresponding to the DGC, CAP and AR sperm preparations from three different semen donors (Figure 1). Differential peptide labeling was performed using Tandem Mass Tag^TM^ 6-plex (TMTsixplex^TM^) Reagents (Thermo Fisher Scientific). The labeling procedure was adapted from the manufacturer’s instructions after several studies conducted in our laboratory (Amaral et al., 2014b; Azpiazu et al., 2014; Bogle et al., 2017; Barrachina et al., 2018). Briefly, proteins were reduced in 9.5 mM tris (2-carboxyethyl) phosphine (TCEP) for 1 h at 55°C, and alkylated with 17 mM iodoacetamide (IAA) for 30 min in the dark. Six-volumes of cold acetone (−20°C) were added and proteins were allowed to precipitate overnight at −20°C. Samples were centrifuged at 17,500 *g* for 10 min, and resuspended in 100 mM triethylammonium bicarbonate (TEAB, Thermo Fisher Scientific) in order to reach a protein concentration of 1 μg/μl. Trypsin was then added at a 1:20 protease-to-protein ratio and the mixture was incubated overnight at 37°C with constant and gentle shaking. Prior to peptide labeling, aliquots were taken from each of the 9 samples and combined in equal amounts to represent the internal control sample. Subsequently, equal amounts of peptides from each sample and the internal control were labeled with TMT isobaric tags. After 1 h of incubation at room temperature, the reaction was quenched with 4 μl of 5% hydroxylamine for 15 min. Labeled peptides from each sample were then combined constituting two different multiplex pools, as indicated in Figure 1: Run 1 with 5 samples (TMT tags 127, 128, 129, 130, and 131) and 1 internal control (TMT tag 126), and Run 2 with 4 samples (TMT tags 127, 128, 129, and 130) and 1 internal control (TMT tag 126). The two multiplex pools containing labeled peptides were dried in a vacuum centrifuge to near dryness and resuspended in 20 μL of 0.5% trifluoracetic acid (TFA) in 5% acetonitrile. Subsequently, peptide purification was conducted by using Pierce C18 Spin Columns (Thermo Fisher Scientific) following manufacturer’s indications.

### Peptide Analysis by LC-MS/MS

Labeled peptides were analyzed via LC-MS/MS with an LTQ-Orbitrap Velos (Thermo-Fisher Scientific) interfaced with an Eksigent nanoLC ultra 2D plus system (AB Sciex, Switzerland). Peptides were injected onto a Pepmap 100 trap column (300 μm × 5 mm, 5 μm, 100 Å) at a flow rate of 400 nL/min. For analytical separation, the trap was switched inline to an Acclaim Pepmap C18 column (75 μm × 15 cm, 3 μm, 100 Å) using a 240 min linear gradient from 5 to 30% acetonitrile in 0.1% formic acid at a flow rate of 400 nL/min. MS/MS analyses were performed using an LTQ Orbitrap Velos (Thermo Fisher Scientific) with a nanoelectrospray ion source. The LTQ-Orbitrap Velos settings included one 30,000 resolution scan for precursor ions followed by MS2 scans of the 20 most intense precursor ions in positive ion mode. MS/MS data acquisition was completed using Xcalibur 2.1 (Thermo Fisher Scientific). The fragmentation method used for identification of TMT labeled peptides was based on higher energy collisional dissociation (HCD) with 40% fixed collision energy (CE).

### LC-MS/MS Raw Data Analysis for Protein Identification and Quantification

LC-MS/MS data were analyzed by Proteome Discoverer 1.4.1.14 (Thermo Fisher Scientific). For database searching, raw MS files were submitted to an in-house *Homo sapiens* UniProtKB/Swiss-Prot 2018 database including *Sus scrofa* trypsin (HUMAN_PIG_Uniprot_Release_2018_03.fasta). SEQUEST HT version 28.0 was used (Thermo Fisher Scientific). Searches were performed using the following settings: 2 maximum miss cleavage sites for trypsin, TMT as a N-Terminal modification, lysine-TMT (+229.163 Da) and methionine-oxidation (+15.995 Da) as dynamic modifications, cysteine-carbamidomethylation (+57.021 Da) as static modification, 20 ppm precursor mass tolerance, 0.6 Da fragment mass tolerance, and 5 ppm peak integration tolerance. The criteria used for protein identification was set as 1% FDR and a minimum of one peptide match per protein. The mass spectrometry proteomics data have been deposited to the ProteomeXchange Consortium via the PRIDE (Perez-Riverol et al., 2019) partner repository with the dataset identifier PXD014871.

Quantitative analysis were performed simultaneously with protein identification using Proteome Discoverer software. For quantification, only unambiguous and unique peptides were considered. For that, the ‘ungrouping’ of proteins from their respective families was used during the quantification process. This avoids the possible ambiguity associated with different isoforms of the same protein (Amaral et al., 2014b; Bogle et al., 2017; Barrachina et al., 2018). The normalized TMT quantification values for each identified spectrum were extracted from the ratio between the reported ion intensities corresponding to each individual sample (TMT-127 to TMT-131) and the reporter ion intensity from the internal control (TMT-126). Quantification values were corrected according to the isotopic purities of the reporter ions provided by the manufacturer.

### Statistical Analysis of Proteomic Data Under Strict Quality Criteria

In order to avoid any chance of false positive findings, robust statistics were performed by applying strict selection criteria on the proteomic data. Specifically, only those proteins with at least 1 unique peptide quantified by ≥2 peptide-to-spectrum matches (PSMs) in all the samples, and with a coefficient of variation < 50% in at least 75% of the samples, were considered for further statistical analyses. This pipeline was set by our group in a previous publication (Barrachina et al., 2018). Statistical analysis were conducted in R (R Development Core Team, 2016) with the package “car” (Fox and Weisberg, 2011). Significant differences among the different sperm lysates in the three donors were evaluated using repeated-measures ANOVA test on log-transformed proteomic quantification values. *P*-values < 0.05 were considered significant if the assumption of sphericity assessed by Mauchly’s test had not been violated. In contrast, Greenhouse–Geisser and Huynh–Feldt corrections were applied when data violated the sphericity assumption. Subsequently, the pairwise *t*-test combined with Holm–Sidak adjustment was applied to identify significant differences between pairs of sample preparations (DGC-CAP, CAP-AR, and DGC-AR). *P*-values < 0.05 after Holm–Sidak adjustment were considered significant. These results were further filtered by applying similar statistical analysis at peptide level, in order to discard those significant proteins whose corresponding individual peptides do not show statistically significant differences between sperm preparations. Protein abundance alterations were considered significant only when corrected *p*-values < 0.05 were found at both protein and peptide levels. GraphPad Prism 7 (GraphPad Software Inc., San Diego, CA, United States) was used for visualizing the results.

### Functional Prediction of the Proteins Detected With Altered Abundance Using Public Databases

In order to predict the functional involvement of the proteins detected with abundance alterations after each sperm preparation (DGC, CAP, and AR), different data were retrieved from the UniProt Knowledgebase^1^ and Pubmed^2^. Gene Ontology (GO) enrichment analyses on terms related to biological processes and cellular components were performed with the Gene Ontology Consortium database^3^ supported by PANTHER v13.1. The significance of the enrichment analyses was calculated by a Fisher’s exact test. *P*-values < 0.05 after FDR adjustment were considered statistically significant.

Gene Ontology Consortium database was also used to retrieve a list of proteins associated to GO terms related to capacitation and acrosome reaction processes. Specifically, the selected GO terms were “capacitation,” “acrosome reaction,” “regulation of acrosome reaction,” “acrosome assembly,” “acrosome matrix dispersal,” “positive regulation of acrosome reaction” and “negative regulation of acrosome reaction,” setting *Homo sapiens* as organism. This list of proteins was submitted, together with the list of altered proteins found in this study, to the String database^4^, in order to explore potential protein-protein interactions and functional association networks between altered proteins and proteins already known to have a role in ejaculated sperm maturation. For String analysis, the confidence threshold was set at 0.7 (high confidence).

Theoretical post-translational modifications (PTMs) in the differentially abundant peptides were predicted using data contained at the PhosphoSitePlus^®^ database^5^.

## Results

### Induction of the Acrosome Reaction With the Calcium Ionophore A23187

**FIGURE 2:**
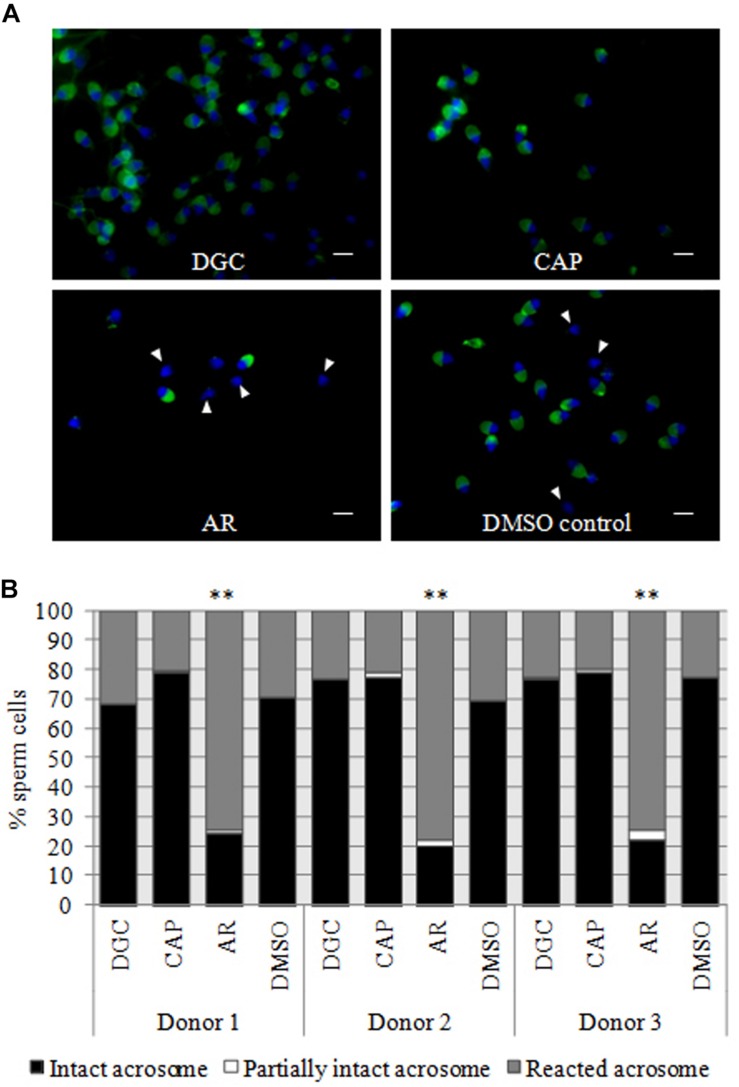
PSA-FITC labeling for the assessment of acrosomal integrity. **(A)** Representative sperm PSA-FITC labeling of one donor sample. PSA-FITC labeling was conducted in purified sperm by density gradient centrifugation (DGC; upper left), sperm incubated with capacitation medium (CAP; upper right) and sperm in which the acrosome reaction was induced by incubation with a calcium ionophore A23187 (AR; lower left). Vehicle control is also shown (DMSO control; lower right). Acrosomal vesicles are shown in green and the sperm cells with reacted acrosomal vesicles are indicated by an arrowhead. Sperm nuclei are stained with DAPI (blue). Scale bar = 5 μM **(B)** Quantification of the number of sperm with an intact acrosome, partially intact acrosome and reacted acrosome in the three fertile donors used for this study. The number of spermatozoa was counted after PSA-FITC labeling on DGC, CAP, AR, and DMSO sperm. Significant differences in the percentage of sperm cells with reacted acrosomes are indicated with stars (*p*-value < 0.01; one-way ANOVA with Holm–Sidak correction).

Before initiating the proteomic analysis, PSA-FITC labeling was performed to assess the integrity of the sperm acrosome in DGC, CAP and AR sperm, as well as to indirectly measure the level of capacitation of the sperm cells (Torabi et al., 2017a). As seen in Figure 2, the absence of signals in the majority of AR sperm (>74%) after exposure to the calcium ionophore A23187 demonstrates that they had mostly undergone acrosomal exocytosis (*p* < 0.01 one-way ANOVA, Holm–Sidak correction). The significant increase in the number of cells with reacted acrosome also indirectly confirmed the efficiency of sperm capacitation. The three samples showed >85% live cells after incubation with calcium ionophore. PSA-FITC staining revealed between 22 and 30% of the cells with a spontaneously reacted acrosome in the DMSO control, which could be considered higher than expected. However, no remarkable changes were found when DMSO cells were compared to DGC or CAP sperm. Taking into account that these levels were equivalent between the three semen samples, no bias on proteomic data was expected.

### Alterations in the Relative Abundance of Proteins After Differential Density Gradient Centrifugation, Incubation in Capacitation Medium and Induction of the Acrosome Reaction

A total of 3658 peptides corresponding to 781 proteins were identified by LC-MS/MS in the lysates from DGC, CAP and AR sperm from the three donor samples (Supplementary Table S2). TMT quantification values were determined for all samples in 1901 peptides from 484 proteins (Supplementary Tables S2, S3), of which 860 peptides derived from 240 proteins met our strict quantification criteria (>1 PSM with <50% variability in >75% of the samples; Supplementary Tables S2, S3). Repeated-measures ANOVA test combined with *post hoc* pairwise *t*-test and Holm–Sidak correction revealed changes in the relative abundance of 48 sperm proteins. However, changes in the levels of albumin (ALB) and semenogelin 2 (SEMG2) were disregarded as they were most likely altered due to technical issues. Certainly, the marked increase of ALB seen in CAP sperm may have been due to the presence of bovine serum albumin in the capacitation medium. In fact, from the three peptides quantified for ALB, the only one not showing significant changes in abundance after incubation with capacitation medium was unique for *Homo sapiens*, while the other two were in common with the bovine sequence (data not shown). Similarly, the lower level of SEMG2 in CAP and AR sperm could be attributed to seminal plasma remnants in DGC sperm that were reduced or even eliminated after the consecutive sperm incubation conditions. Of note, no more proteins were found showing differences with a magnitude similar to SEMG2, which would discard additional artifactual extraneous contamination.

Relative inter-lysate changes in protein abundance were further validated by conducting quantitative analysis at the peptide level. This strategy revealed that peptides from some of the 48 proteins did not show significant differences following sequential incubation conditions. After *p*-value correction at both the peptide and protein levels, only 36 proteins showed significant inter-lysate differences and were considered for further analyses (Table 1 and Supplementary Table S4). Of note, all these proteins have been identified in the human sperm cell by previous proteomic studies (Castillo et al., 2018). GO enrichment terms showed that this subset of 36 proteins was mainly involved in fertilization-related processes, including “fertilization,” “sperm-egg recognition,” and “acrosome reaction,” and energy production-related processes, such as “ATP metabolic process” and “glycolytic process,” among others (*p* < 0.05 after FDR correction; Table 2). Regarding localization in the sperm cell itself, the 5 most enriched cellular components were “sperm flagellum,” “acrosomal vesicle,” “mitochondrial proton-transporting ATP synthase,” “acrosomal membrane,” and “outer dense fiber” (*p* < 0.05 after FDR correction; Table 2).

**TABLE 1:** Quantified proteins with an altered abundance after the consecutive incubation with capacitation medium and *in vitro* induction of the acrosome reaction.

**Gene name**	**# Peptides quantified under** **strict criteria**	**#Peptide differentially** **abundant**	**DGC-CAP/CAP-AR/** **DGC-AR**	**Abundance** **alteration**	**Related** **function**
ACR	10	4	DGC-CAP; CAP-AR	↓	Fertilization
ACRBP	17	8	CAP-AR	↓	Fertilization
ACRV1	3	3	DGC-CAP; CAP-AR	↓	Fertilization
AKAP3	16	12	DGC-CAP	↓	Sperm motility
AKAP4	22	16	DGC-CAP	↓	Sperm motility
ATP5F1A	15	1	DGC-CAP	↑	Energy production
ATP5F1D	2	2	CAP-AR	↑	Energy production
ATP5IF1	3	1	DGC-AR	↑	Energy production
ATP5O	1	1	DGC-AR	↑	Energy production
CCT2	2	1	DGC-AR	↓	Protein folding
ENO1	11	1	DGC-AR	↓	Energy production
ERLIN2	1	1	DGC-AR	↑	Signaling
FAM209A	2	1	DGC-CAP	↑	Unknown
FAM71B	2	1	DGC-AR	↓	RNA biogenesis
GGH	5	2	CAP-AR	↓	Metabolism of folic acid
GLUL	3	1	DGC-CAP	↓	Signaling
GSTM3	9	3	DGC-AR	↓	Detoxification
HK1	19	2	CAP-AR	↓	Energy production
HSPA2	18	3	DGC-AR	↓	Protein folding
LYZL4	3	1	DGC-AR	↓	Fertilization
LYZL6	1	1	DGC-CAP	↓	Fertilization
ODF2	12	5	DGC-CAP	↓	Sperm motility
PGAM2	6	1	CAP-AR	↓	Sperm motility
PGK2	14	5	DGC-CAP	↓	Sperm motility
PHB2	1	1	DGC-CAP; CAP-AR	↑	Signaling
PSMA4	1	1	CAP-AR	↑	Protein degradation
REEP5	1	1	CAP-AR	↑	Signaling
ROPN1B	1	1	DGC-AR	↓	Sperm motility
RSPH1	1	1	DGC-AR	↑	Sperm motility
SLC2A14	1	1	DGC-AR	↓	Detoxification
SOD2	1	1	DGC-AR	↑	Detoxification
SPACA3	5	2	DGC-CAP	↓	Fertilization
SPACA7	1	1	CAP-AR	↓	Fertilization
TMED10	3	1	CAP-AR	↑	Vesicular trafficking
TUBA1A	1	1	DGC-CAP	↑	Sperm motility
ZPBP	10	2	CAP-AR	↓	Fertilization

*DGC-CAP: proteins differentially detected after incubation with capacitation medium; CAP-AR: proteins differentially detected after induction of the acrosome reaction; DGC-AR: proteins differentially detected after incubation of capacitation medium followed by the induction of the acrosome reaction; ↑: increased abundance; ↓: decreased abundance; Related function: according to public databases.*

**TABLE 2:** Gene Ontology (GO) terms enrichment analysis.

**GO term**	**Genes**	**FDR**
**GO biological process**		
Fertilization	11	1.34E-10
ATP metabolic process	7	1.42E-04
Glycolytic process	4	1.62E-03
Sperm-egg recognition	4	2.37E-03
Acrosome reaction	3	5.30E-03
Spermatogenesis	7	1.15E-02
Gluconeogenesis	3	2.18E-02
Inner mitochondrial membrane organization	3	2.74E-02
**GO cellular component**		
Sperm flagellum	8	5.84E-09
Acrosomal vesicle	8	8.16E-09
Mitochondrial proton-transporting ATP synthase complex	3	1.21E-03
Acrosomal membrane	3	1.28E-03
Extracellular vesicle	13	1.85E-03
Outer dense fiber	2	6.75E-03
Meiotic spindle	2	1.62E-02
Nucleus	23	1.71E-02
Sperm principal piece	2	3.91E-02

*GO terms corresponding to biological processes and cellular components that are significantly enriched in the set of sperm proteins with altered abundance after incubation with capacitation medium and induction of the acrosome reaction. *P*-value < 0.05 after FDR correction.*

**FIGURE 3:**
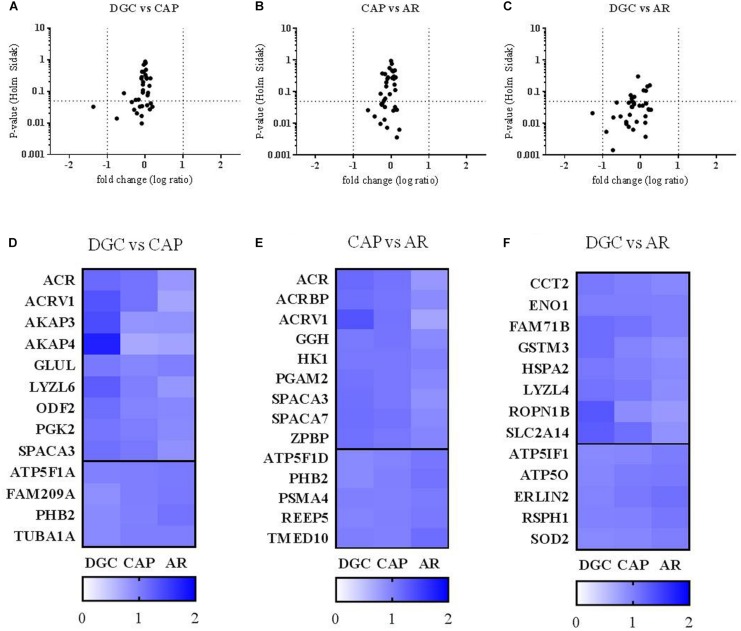
Differences in the abundance of sperm proteins after incubation with capacitation medium and *in vitro* induction of the acrosome reaction. **(A–C)** *p*-value (repeated-measures ANOVA with Holm–Sidak correction) and fold changes (log ratio of TMT intensities of each sample with the internal control) for all quantified proteins (under strict criteria) after incubation with sperm capacitation medium (DGC vs. CAP), induction of the acrosome reaction (CAP vs. AR) and the combination of both treatments (DGC vs. AR). Fold change of –1 represents reduction to half, and +1 increasing to double (vertical dot lines). Horizontal dotted lines are set at *p*-value = 0.05. **(D–F)** heatmaps showing the ratio of TMT intensities from each sample with the internal control corresponding to proteins with statistically significant differences in their abundance after the different sperm treatments (DGC, CAP, and AR). Proteins with altered abundance were grouped depending on the treatment leading to the significant differences (DGC vs. CAP, CAP vs. AR, DGC vs. AR). Horizontal thick line separates proteins decreasing in abundance after the treatment (top) from those detected with higher protein levels (bottom).

Looking more closely at the 36 proteins with altered inter-lysate concentrations, 13 differed significantly between DGC and CAP sperm (9 with decreased and 4 with increased protein levels in CAP sperm lysates; Figures 3A–D and Supplementary Figure S1), and 13 differed significantly between DGC and AR sperm (8 with reduced abundance and 5 with increased abundance in AR sperm lysates; Figures 3C–F and Supplementary Figure S2). Interestingly, the relative abundance of 14 proteins was only altered after induction of the acrosome reaction (AR sperm; Figures 3B–E). Of those, 9 proteins showed lower and 5 higher levels of abundance in AR lysates (Figure 3E and Supplementary Figure S3).

### Functional Roles of Sperm Proteins Differentially Solubilized After Consecutive Incubation in Capacitation Medium and Following Induction of the Acrosome Reaction

**FIGURE 4:**
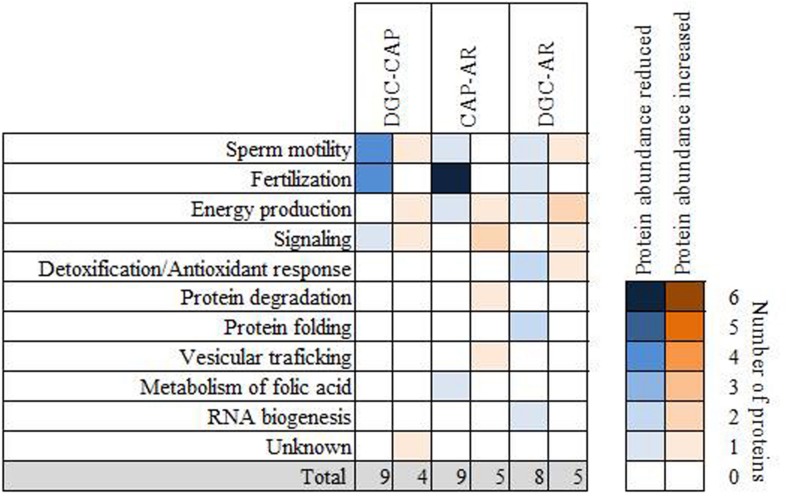
Functional involvement of proteins detected with an altered abundance after incubation with capacitation medium and *in vitro* induction of the acrosome reaction. The results are based on the data contained in the public databases Uniprot KB and Pubmed. Proteins present at reduced abundance are shown in blue and proteins detected with increased abundance in orange. The intensity of the color increases according to the number of proteins related to each functional category. DGC-CAP: changes after incubation with capacitation medium. CAP-AR: changes after induction of the acrosome reaction; DGC-AR: changes after synergic effect of incubation with capacitation medium followed by induction of the acrosome reaction.

The small number of proteins with altered levels of abundance identified for each of the sperm preparation groups limited the usefulness of GO enrichment analysis for identifying potential functional roles of these proteins. Instead, this was evaluated by searching in public databases and close examination of the available literature. Eleven functional categories were identified in this way, including “sperm motility,” “fertilization,” “energy production,” “signaling,” “detoxification/antioxidant response,” “protein degradation,” “protein folding,” “vesicular trafficking,” “metabolism of folic acid,” “RNA biogenesis” and “unknown.” Figure 4 shows the distribution of proteins in each of the functional groups according to the stage of post-ejaculatory sperm processing. The specific category for each protein is indicated in Table 1.

Following incubation of the DGC sperm in capacitation medium, differences in their relative abundance were detected in proteins involved in sperm motility, fertilization, energy production and signaling (Figure 4). Induction of the acrosome reaction resulted in the reduction of the abundance of proteins involved mainly in fertilization, which includes sperm-oocyte recognition, binding and fusion (Figure 4). In addition, reduced levels of proteins with known roles in sperm motility, energy production and metabolism of folic acid were also identified in AR lysates (Figure 4 and Table 1). Interestingly, proteins with increased relative abundance following the acrosome reaction were related to energy production, signaling, protein degradation and vesicular trafficking (Figure 4). Regarding differences observed between DGC and AR groups, which tests for changes induced by the combination of both incubation in capacitation medium and the acrosome reaction on DGC sperm, proteins with altered abundance related to detoxification/antioxidant response, protein folding and RNA biogenesis were also revealed (Figure 4).

To further explore the potential functional roles of the lysate proteins with altered post-processing abundance or possible synergies between them, protein-protein interactions between these proteins and all proteins associated to GO terms related to capacitation or acrosome reaction were assessed using the STRING database. This analysis highlighted the interaction of altered proteins not previously related to these processes with other proteins known to play important roles in the maturation of ejaculated sperm (Table 3 and Supplementary Figure S4). For example, ERLIN2, which differed in abundance between DGC and AR conditions, is known to interact with CFTR, a protein associated with the GO term for capacitation. Likewise, STRING analysis revealed an interaction between ATP5F1A (also known as ATP5A), the relative abundance of which was altered following incubation in CAP medium, with DLD, a protein related with the GO of “capacitation” (Table 3 and Supplementary Figure S4).

**TABLE 3:** Protein–protein interactions between altered proteins detected in this study and proteins associated to GO terms of capacitation and acrosome reaction.

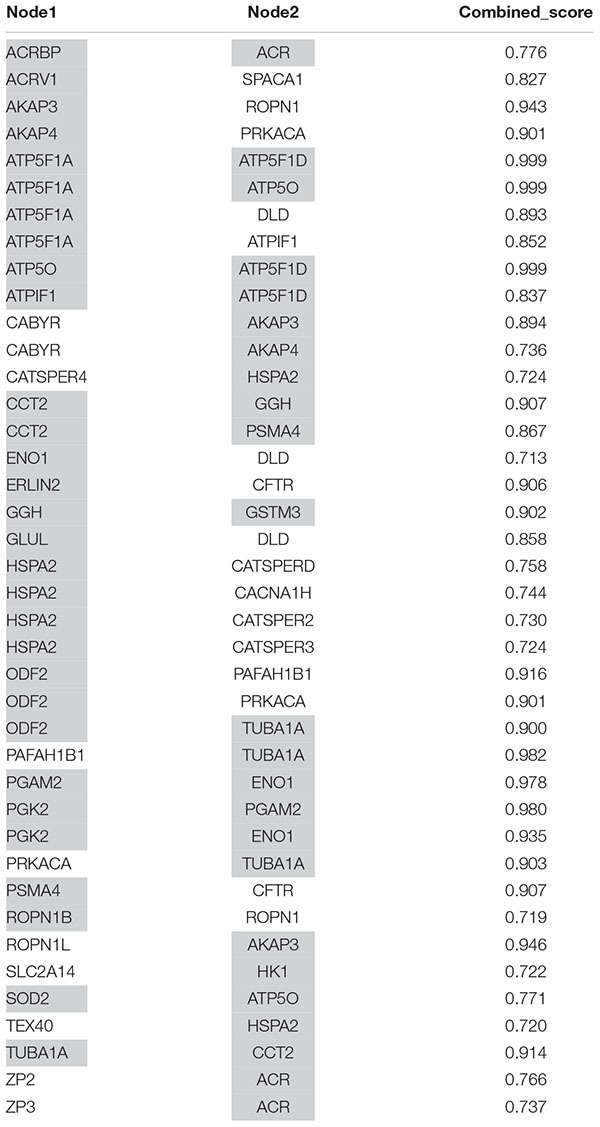

*Proteins found with altered abundance in this study are highlighted in gray. Confidence score > 0.7 is set as high by STRING database.*

### Role of PTMs and Isoforms in Protein Abundance Alterations Detected After Incubation in Capacitation Medium and Following Induction of the Acrosome Reaction

**FIGURE 5:**
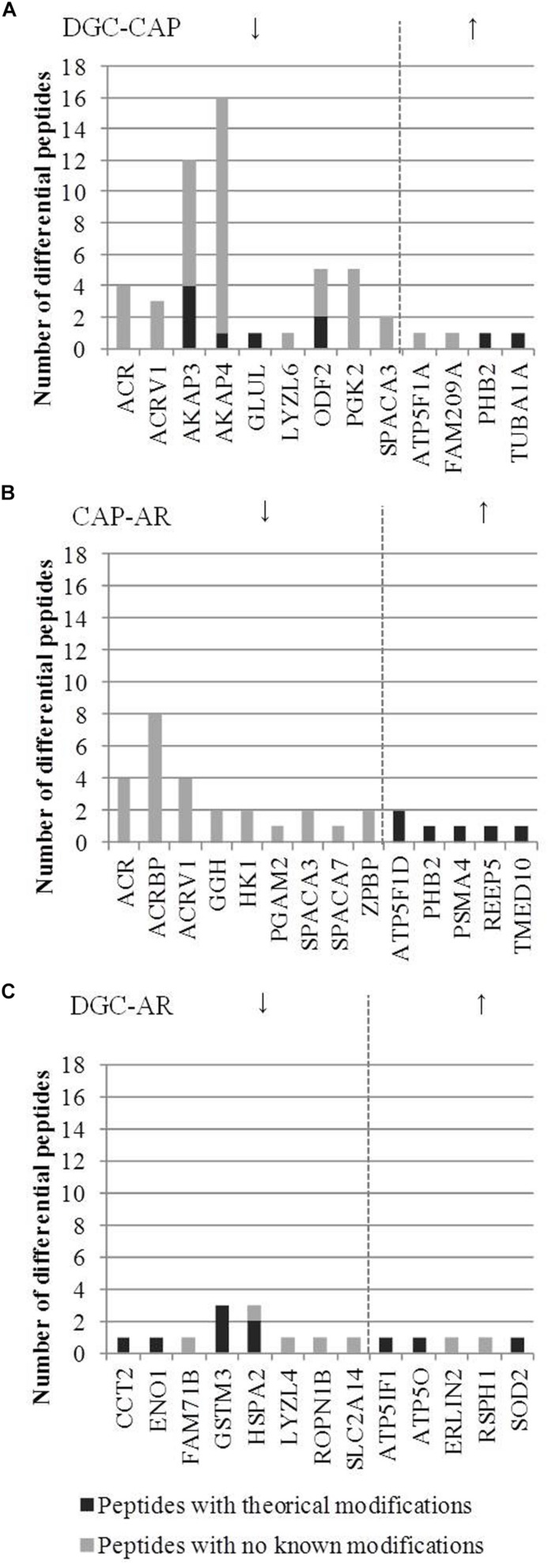
Altered peptides with potential post-translational modifications corresponding to proteins identified with significantly different abundance after incubation with capacitation medium and *in vitro* induction of the acrosome reaction. **(A)** proteins found with altered abundance after incubation with capacitation medium. **(B)** proteins found with altered abundance after induction of the acrosome reaction. **(C)** proteins found with altered abundance after the synergic effect of incubation in capacitation medium followed by induction of the acrosome reaction. Arrows indicate whether the abundance of the proteins was increased or decreased after incubation with capacitation medium and *in vitro* induction of the acrosome reaction.

To identify a possible mechanism behind the relative changes in protein abundance following the consecutive incubation of sperm with capacitation medium and the induction of the acrosome reaction, the presence of PTMs in residues from peptides with significant inter-lysate differences in relative abundance was explored by using data from the PhosphoSitePlus^®^ database. Potentially phosphorylated, acetylated, ubiquitinated, succinylated and sumoylated residues were identified (Figure 5 and Supplementary Table S4). Interestingly, no PTMs were described for the differential peptides identified for proteins with reduced abundance following induction of the acrosome reaction (Figure 5B). However, retrieved data revealed modified residues for all the significantly altered peptides corresponding to proteins with an increased relative abundance in AR lysates (Figure 5B). Described PTMs were also attributed to peptides corresponding to altered proteins after sperm incubation in capacitation medium, or after consecutive incubation with capacitation medium and following induction of the acrosome reaction (Figures 5A–C).

In addition to the above observations, assignment of the peptides with altered abundance to specific protein isoforms was also undertaken. Three peptides were quantified for protein ATP5IF1, which showed increased protein levels only in comparison between DGC and AR sperm lysates. Following statistical analysis, the only peptide shown to differ significantly was exclusive for the ATP5IF1 isoform 1 (Table 1 and Supplementary Table S4).

## Discussion

Studying the molecular processes that govern how ejaculated spermatozoa mature in the female genital tract and become competent to fertilize the oocyte is now a key component of basic research in the field of Andrology. The fundamental cellular and molecular mechanisms and their components driving capacitation and acrosome reaction processes, however, are not yet completely understood. Identifying sperm components displaying alterations in abundance during *in vitro* post-ejaculated sperm maturation could aid to unravel hidden molecular aspects which can impact on male fertility, and the use of novel high-throughput molecular approaches is a good strategy to help achieve this goal.

There are published reports that made use of MS to help identify protein differences before and after the induction of sperm capacitation in human and model species (Ficarro et al., 2003; Bailey et al., 2005; Sleight et al., 2005; Secciani et al., 2009; Baker et al., 2010; Zigo et al., 2013; Kwon et al., 2014; Wang J. et al., 2015; Hernández-Silva et al., 2019; Hou et al., 2019). However, the number of studies focusing on human sperm and using the most advanced techniques is limited. In addition, to the best of our knowledge, acrosomal exocytosis has not yet been evaluated from a proteomic perspective. In the present study, we applied, for the first time, a high-throughput proteomic procedure combined with isotopic peptide labeling to help characterize not only changes in the proteomic profile of healthy spermatozoa after *in vitro* incubation with capacitation medium, but also after the release of the acrosomal contents. It is important, however, to be mindful of the fact that human semen samples are biologically very heterogeneous and that among the sperm population, only a few sperm will be capable of becoming competent to fertilize the oocyte. Both intra- and inter-individual sample variance might influence the results of ‘omics- based research and, therefore, setting strict criteria for data analysis becomes essential to improve the impact of these data (Lualdi and Fasano, 2019). In the current study, we applied robust statistics at both the protein and peptide levels and considered potential technical bias. This workflow led us to identify 36 sperm proteins with a remarkably altered abundance in sperm lysates as a consequence of the incubation with capacitation medium, the calcium ionophore-induced acrosome reaction or both joint processes.

In order to understand whether the apparent differences in protein abundance are truly reflective of novel factors that may be critical during the acquisition of sperm fertilization capacity, it is necessary to consider their potential causes and effects. This is highly relevant since spermatozoa are inactive at both the transcriptional and the translational levels, excluding the possibility of increased gene expression accounting for rises in the relative abundance of some proteins in sperm lysates and placing more emphasis on dynamic molecular changes due, perhaps, to protein modification, degradation or translocation (Signorelli et al., 2012; Jodar et al., 2013; Amaral et al., 2014a; Castillo et al., 2018). We found that incubation with capacitation medium induced quantitative changes in proteins involved mainly in sperm motility and signaling, which are processes regulated by PTMs. Indeed, it is widely thought that important cellular changes facilitating sperm motility during capacitation is mediated by tyrosine phosphorylation through protein kinase A (PKA) activation and the down-regulation of Ser/Thr phosphatases by Src family kinases (Ficarro et al., 2003; Visconti et al., 2011; Battistone et al., 2013; Signorelli et al., 2013; Wang J. et al., 2015). The A kinase anchoring proteins (AKAPS) are actively involved in PKA-dependent protein tyrosine phosphorylation, and a decrease in the relative inter-lysate abundance of AKAP3 and AKAP4 was found in the current study after incubation with capacitation medium. A requirement for AKAP3 degradation in sperm capacitation has been reported (Hillman et al., 2013; Vizel et al., 2015), which supports our results. Likewise, we found relative alterations in other proteins known to be involved in PKA-dependent signaling processes, including ROPN1B (Carr et al., 2001; Fiedler et al., 2013) and the acquisition of sperm motility, including PGK2 (Danshina et al., 2010; Shen et al., 2013; Liu et al., 2016; Huang et al., 2017), ODF2 (Huang et al., 2015; Wang X. et al., 2015; Zhao et al., 2018), and RSPH1 (also known as TSGA2) (Hui et al., 2006; Shetty et al., 2007; Onoufriadis et al., 2014).

While several proteomic reports can be found focused on sperm capacitation, this is the first study applying advanced proteomic strategies to analyze protein changes following the release of the acrosomal content. It is important to take into consideration that, although calcium influx is required to initiate the process, the use of a calcium ionophore does not mimic the natural trigger of the acrosome reaction, which requires binding to the zona pellucida (Liu and Baker, 1996a, b). However, tests simulating a zona-mediated physiological acrosome reaction are restricted by the limited availability of enough biological material for proteomic studies. Calcium ionophore A23187 is therefore an acceptable alternative when aiming to describe protein changes, since the results in terms of the quantitative loss of acrosomal contents should be equivalent. We found 9 proteins with a lower abundance after the acrosome reaction compared with lysates from CAP sperm, while 5 proteins seemed to increase their relative protein levels. Of note, none of the peptides from proteins with a lower abundance after the acrosome reaction contained residues with potential PTMs in their sequence. In contrast, all proteins with relatively higher abundance in AR lysates could be targets of protein modifiers. These results suggest that all proteins with apparently lower abundance in AR lysates may be released or relocated during the acrosome reaction, while those with relative increases in abundance may represent modified proteins involved in signaling processes that have lost the corresponding PTM. In support of this hypothesis, many of the proteins with diminished protein levels after induction of the acrosome reaction are known to be components of the acrosomal vesicle, including ACR, ACRBP, ARV1, and SPACA7 and, therefore, those might be released together with the acrosomal content. However, potential changes in the distribution of sperm proteins should also be considered, as it has been observed in previous studies using a number of different methods (Torabi et al., 2017a). The lysis buffer used in this study to solubilize sperm proteins most likely gained access to compartments of the sperm during and following capacitation that were hidden beforehand. A similar effect would lead to the release of proteins following the acrosome reaction where the acrosomal vesicle is lost. This does not imply that our data is a technical artifact (notwithstanding the status of albumin and semenogelin, both disregarded); instead, we propose that it is mainly the dynamic changes in cellular and structural aspects of the sperm occurring during capacitation and the acrosome reaction that is the main driver of these results, although active enzymatic destruction of sperm proteins during these processes may also contribute. It should also be recognised that successive transitional stages take place before the complete release of the acrosome contents (Buffone et al., 2008). Therefore, further studies considering different time points during the acrosome reaction would shed light into the functional involvement of these proteins.

The finding of alterations in the abundance of sperm proteins already known to be involved in sperm capacitation and the acrosome reaction supports the reliability of the strategy followed by this study. Moreover, it also increases the interest in those (altered) proteins never associated previously with sperm functionality. For example, our results suggest that, in our *in vitro* conditions, sperm maturation induced changes in the ER lipid raft-associated 2 (ERLIN2) protein, which is known to mediate degradation of inositol 1,4,5-triphosphate receptors (Wang Y. et al., 2009; Wright et al., 2018). Interestingly, STRING analysis revealed the interaction of ERLIN2 with the cystic fibrosis transmembrane conductance regulator protein (CFTR), which is essential during sperm capacitation through PKA-dependent phosphorylation, alkalization and hyperpolarization (Puga Molina et al., 2017, 2018b). The reduction in the abundance of gamma-glytamyl hydrolase (GGH) in AR sperm is also noteworthy. The interaction analysis revealed the association of GGH with the T-complex protein 1 subunit beta (CCT2), which is a chaperone involved in sperm-ZP binding (Redgrove et al., 2011). Only an involvement of GGH in the metabolism of folic acid is reported in the literature (DeVos et al., 2008). However, since both GGH and CCT2 showed significant alterations in protein abundance after the process of acrosome reaction, GGH is suggested as a potential candidate for further study. In addition, GGH might also interact with Glutathione S-transferase Mu 3 protein (GSTM3), which has been previously related to capacitation and ZP binding using MS (Ficarro et al., 2003; Petit et al., 2013). Another novel protein thought to be involved in the process of the acrosome reaction is the Transmembrane em24 domain-containing protein 10 (TMED10), which, although not previously associated with sperm function, may be involved in vesicular trafficking in other cellular systems (Nakano et al., 2017; Luteijn et al., 2019).

Sperm capacitation and the acrosome reaction are complex processes involving many proteins and signaling pathways. Indeed, the sheer complexity precludes our obtaining a complete proteomic picture of these processes in just one MS-based study. A combination of data from high-throughput studies applying different approaches, such as enrichment in peptides with specific PTMs, the conduction of cell fractionation or the use of isobaric tags for protein quantification, among others, is required (Amaral et al., 2014a; Codina et al., 2015). In this study, we have applied TMT labeling to quantify changes in sperm proteins in response to stimuli leading to an *in vitro* simulation of post-gonadal sperm maturation. We believe that our results increase the current knowledge about maturation of ejaculated sperm and suggest new players in the process. However, further experiments at peptide level are now required. By dissected the process of capacitation and the acrosome reaction at the peptide level, there is the possibility of understanding the roles of these proteins during the acquisition of competence to fertilize the oocyte and to confirm both the presence of modifications and alterations thereof in infertile patients. In addition, our results highlight the limitations of the currently available *in vitro* methods to mimic the *in vivo* situation. Capacitation and the acrosome reaction occur within the female reproductive system and thus, secretions from and components of the female tract may have an impact on sperm acquisition of fertilizing capacity. Therefore, the development of 3-D culture systems that mimic the oviduct and provide an *in vivo-*like environment to the sperm cell are desirable (Ferraz et al., 2017). A better understanding of the molecular mechanism mediating fertilization is essential in order to improve current infertility diagnosis and treatment.

## Data Availability Statement

The datasets generated for this study can be found in the ProteomeXchange Consortium via the PRIDE partner repository, dataset identifier PXD014871.

## Ethics Statement

The studies involving human participants were reviewed and approved by Clinical Research Ethics Committee of the Hospital Clínic of Barcelona. The donors provided their written informed consent to participate in this study.

## Author Contributions

OB, FT, and RO designed the study. JB was involved in donor samples collection. JC, OB, and FT performed the research. JE performed the proteomic technology. JC, MJ, FT, and DD-D analyzed the data. JC, MJ, and RO interpreted the data. JC, MJ, FT, DM, and RO drafted the manuscript. All authors critically reviewed and approved the final version of the manuscript.

## Conflict of Interest

The authors declare that the research was conducted in the absence of any commercial or financial relationships that could be construed as a potential conflict of interest.

## Footnotes

1 https://www.uniprot.org/

2 https://www.ncbi.nlm.nih.gov/pubmed

3 http://geneontology.org/

4 https://string-db.org/

5 https://www.phosphosite.org

## Supplementary Material

FIGURE S1 Proteins detected with altered abundance after incubation of the sperm cells with capacitation medium (DGC-CAP). The mean of the ratio between TMT intensities of the three donors and the internal control are shown for each protein at each sperm condition.

FIGURE S2 Proteins detected with altered abundance after the combination of sperm incubation with capacitation medium and induction of the acrosome reaction (DGC-AR). The mean of the ratio between TMT intensities of the three donors and the internal control are shown for each protein at each sperm condition.

FIGURE S3 Proteins detected with altered abundance after the induction of the acrosome reaction (CAP-AR). The mean of the ratio between TMT intensities of the three donors and the internal control are shown for each protein at each sperm condition.

FIGURE S4 Protein–protein interaction networks between proteins found with altered abundance in this study and all those proteins associated with Gene Ontology terms related to capacitation and acrosome reaction. Proteins known to be functionally associated to the processes of capacitation and acrosome reaction were retrieved from the Gene Ontology Consortium database and submitted to STRING database together with the list of proteins found in this study with statistical significant differences in abundance. Only those protein-protein interactions with a high confidential score (>0.7) are shown.

TABLE S1 Seminal parameters of the semen samples included in the study and obtained from 3 fertile donors.

TABLE S2 Proteins identified in the three sperm donors and three sperm conditions by LC-MS/MS. The number of identified peptides, PSMs at both TMT runs, quantified peptides in all samples and quantified peptides that fit the strict selection criteria for statistics are indicated.

TABLE S3 Peptides identified by LC-MS/MS containing quantification values.

TABLE S4 Proteins identified with altered abundance. *P*-values after one way ANOVA for repeated measures and *t*-test with Holm–Sidak adjustment are provided for both proteins and peptides. *P*-values <0.05 are highlighted in green.
