# Supplementary figures and images for: Proteomic Changes in Human Sperm During Sequential in vitro Capacitation and Acrosome Reaction
Source: Front Cell Dev Biol. 2019 Nov 20;7:295. doi: 10.3389/fcell.2019.00295 (PMC6879431; doi:10.3389/fcell.2019.00295)

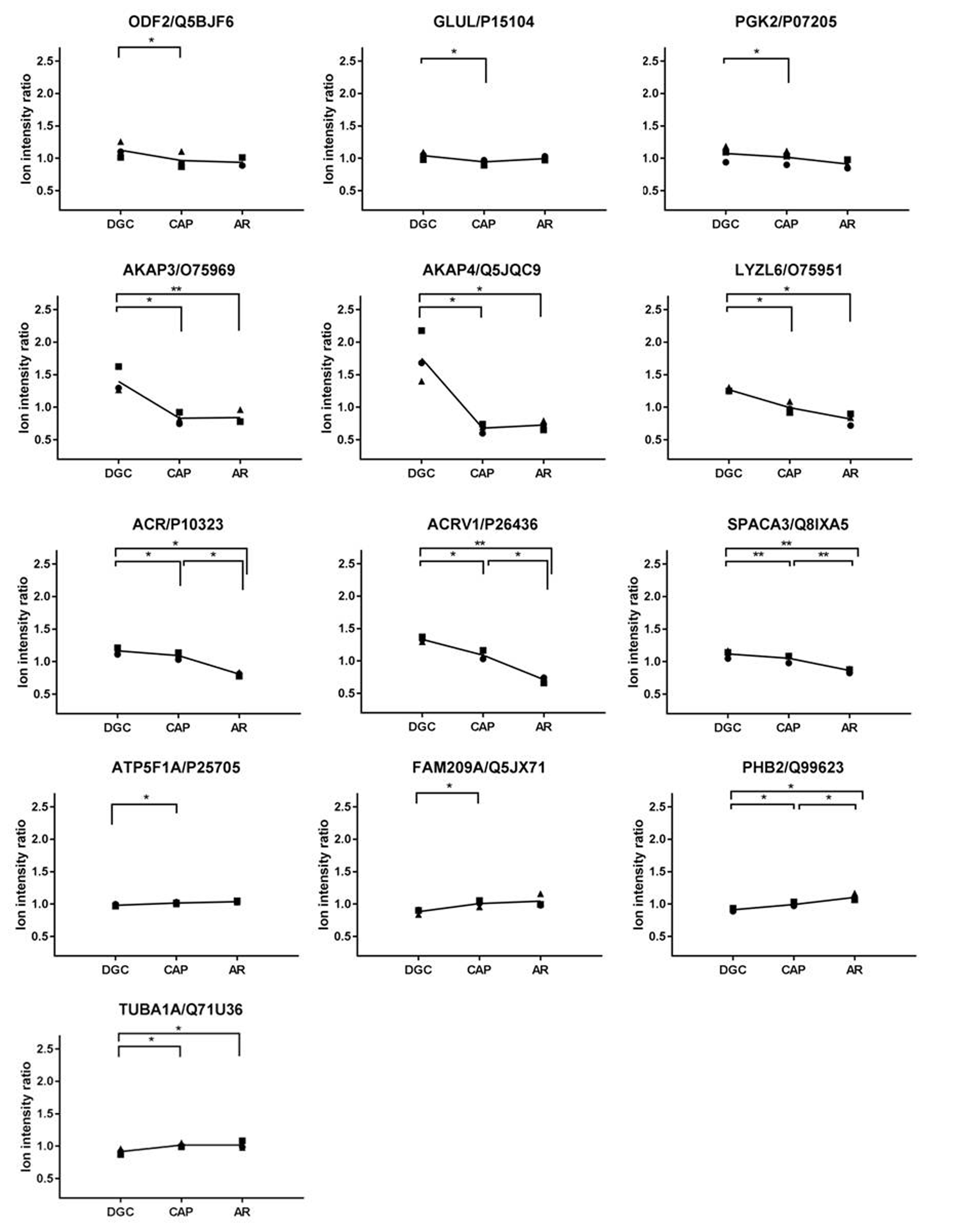

Supplement: FIGURE S1 — Proteins detected with altered abundance after incubation of the sperm cells with capacitation medium (DGC-CAP). The mean of the ratio between TMT intensities of the three donors and the internal control are shown for each protein at each sperm condition. [file Image_1.JPEG]

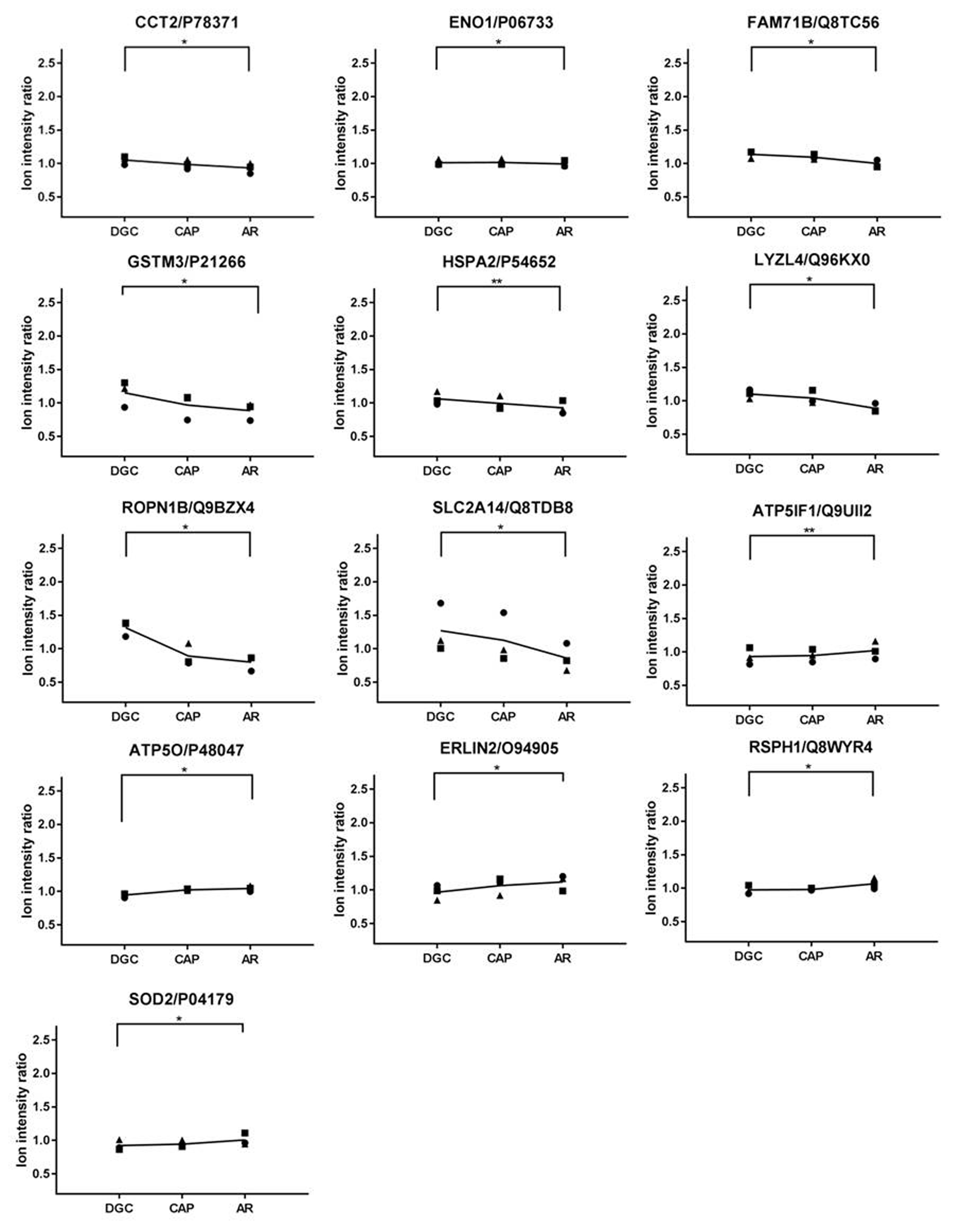

Supplement: FIGURE S2 — Proteins detected with altered abundance after the combination of sperm incubation with capacitation medium and induction of the acrosome reaction (DGC-AR). The mean of the ratio between TMT intensities of the three donors and the internal control are shown for each protein at each sperm condition. [file Image_2.JPEG]

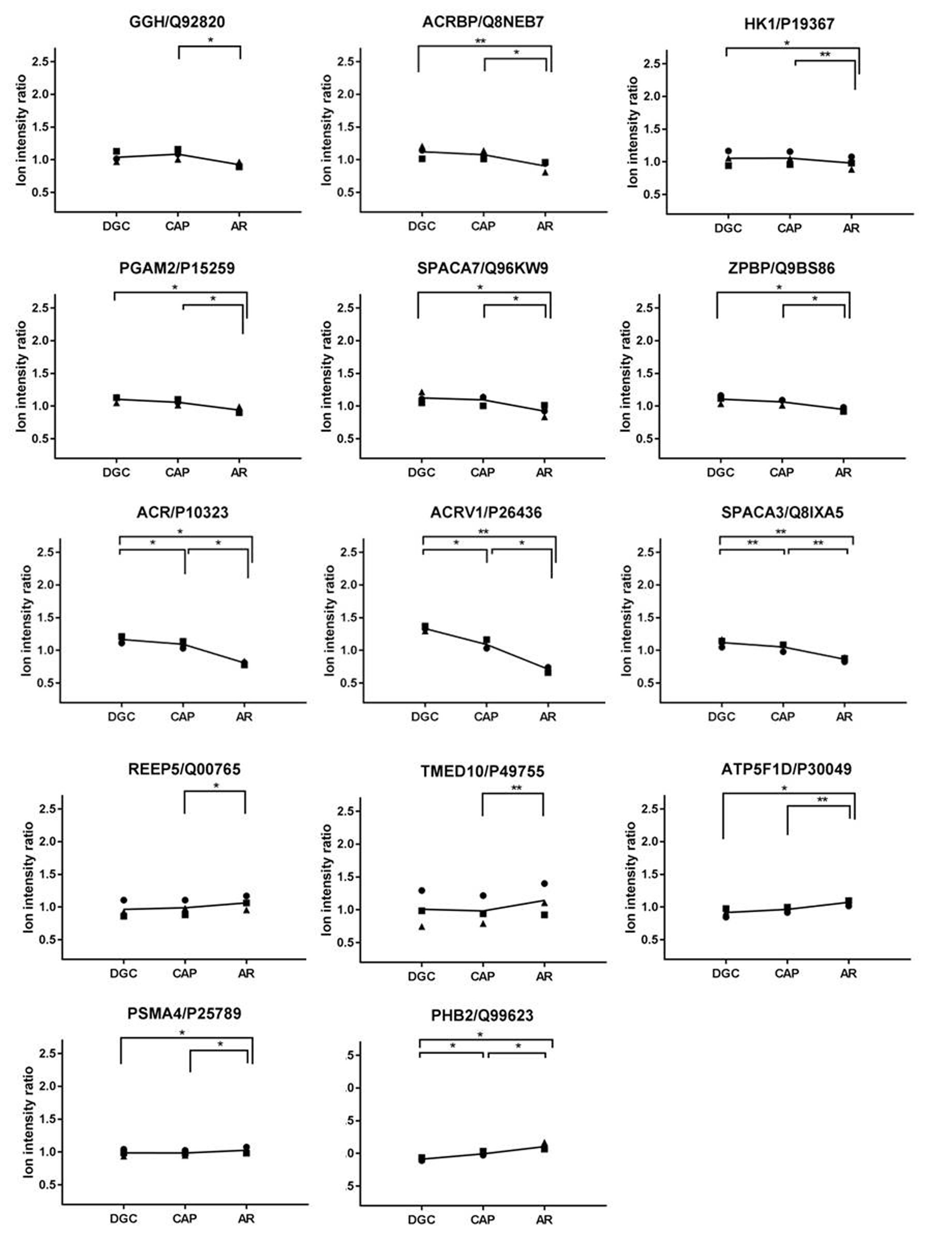

Supplement: FIGURE S3 — Proteins detected with altered abundance after the induction of the acrosome reaction (CAP-AR). The mean of the ratio between TMT intensities of the three donors and the internal control are shown for each protein at each sperm condition. [file Image_3.JPEG]

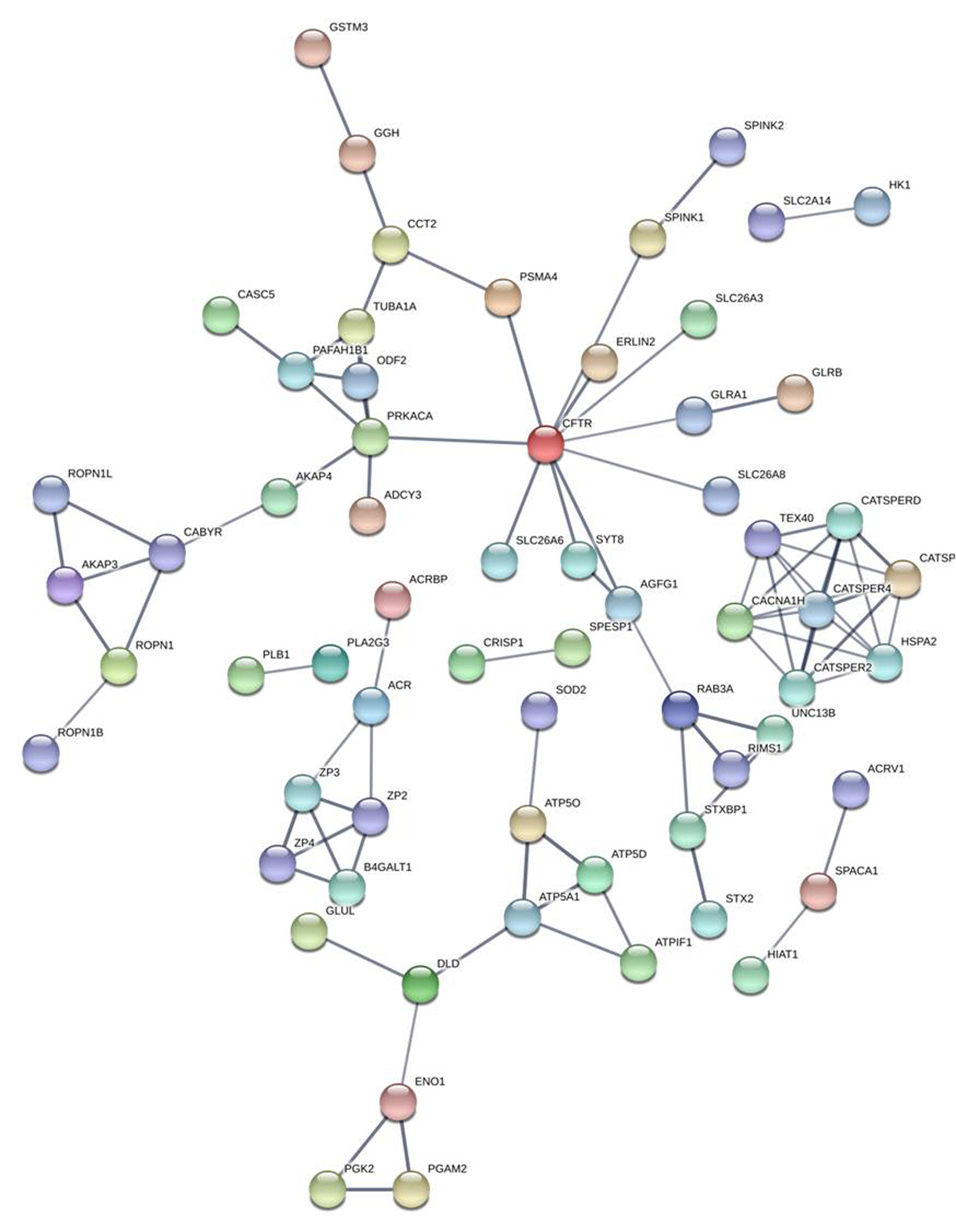

Supplement: FIGURE S4 — Protein–protein interaction networks between proteins found with altered abundance in this study and all those proteins associated with Gene Ontology terms related to capacitation and acrosome reaction. Proteins known to be functionally associated to the processes of capacitation and acrosome reaction were retrieved from the Gene Ontology Consortium database and submitted to STRING database together with the list of proteins found in this study with statistical significant differences in abundance. Only those protein-protein interactions with a high confidential score (>0.7) are shown. [file Image_4.JPEG]
